# Possible therapeutic effect of orally administered ribavirin for respiratory syncytial virus-induced acute respiratory distress syndrome in an immunocompetent patient: a case report

**DOI:** 10.1186/s13256-017-1514-x

**Published:** 2017-12-20

**Authors:** Byung Woo Yoon, Seung Hyeun Lee

**Affiliations:** 1 Department of Internal Medicine, Hanil General Hospital, Seoul, Republic of Korea; 2 Department of Internal Medicine, Kyung Hee University School of Medicine, Seoul, Republic of Korea; 3 Department of Pulmonary and Critical Care Medicine, Kyung Hee University School of Medicine, Kyungheedae-ro 23, Dongdaemun-gu, Seoul, 02447 Republic of Korea

**Keywords:** Case report, Respiratory syncytial virus, Ribavirin, Acute respiratory distress syndrome

## Abstract

**Background:**

Human respiratory syncytial virus usually causes self-limiting upper respiratory infection and occasionally causes pneumonia in immunocompromised hosts. Respiratory syncytial virus-induced severe pneumonia or acute respiratory distress syndrome in immunocompetent adults has been rarely described. Unfortunately, optimal treatment has not been established for this potentially fatal condition. We report a case of respiratory syncytial virus-induced acute respiratory distress syndrome occurring in a previously healthy man successfully treated with orally administered ribavirin.

**Case presentation:**

An 81-year-old previously healthy Korean man presented with cough, dyspnea, and febrile sensation. He had hypoxemia with diffuse ground glass opacity evident on chest radiography, which progressed and required mechanical ventilation. All microbiological tests were negative except multiplex real-time reverse transcriptase polymerase chain reaction using respiratory specimen, which was positive for human adenovirus. Under the diagnosis of respiratory syncytial virus-induced acute respiratory distress syndrome, orally administered ribavirin was administered and he recuperated completely without complications.

**Conclusion:**

This case demonstrates the potential usefulness of orally administered ribavirin as a therapeutic option for severe respiratory syncytial virus infection, at least in an immunocompetent host.

## Background

Human respiratory syncytial virus (RSV) is an enveloped ribonucleic acid (RNA) virus belonging to the family *Paramyxoviridae*. RSV is a major pathogen causing lower respiratory tract infection in babies and young children, leading to hospitalization and death [1]. In adults, it usually causes upper respiratory infection that is self-limiting. However, with recent increases in hematopoietic stem cell and solid organ transplantation, RSV infection is attracting clinical attention as a key pathogen of opportunistic infections which is associated with high mortality and morbidity [2]. RSV-induced severe pneumonia or acute respiratory distress syndrome (ARDS) in immunocompromised patients is not uncommon. However, it has been rarely described in immunocompetent adults. Here we report a case of ARDS due to RSV occurring in a previously healthy adult successfully treated with orally administered ribavirin.

## Case presentation

**Fig. 1:**
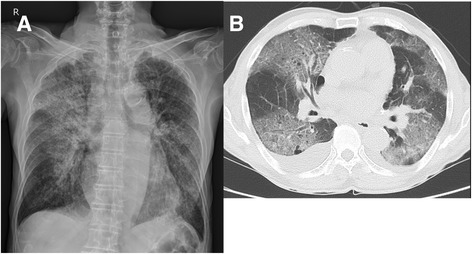
Chest imaging at admission. Chest X-ray showing diffuse haziness which is dominant in right lung (**a**). Chest computed tomographic scan showing ground glass opacities in both lungs with pleural effusion dominant at the right hemithorax (**b**)

**Fig. 2:**
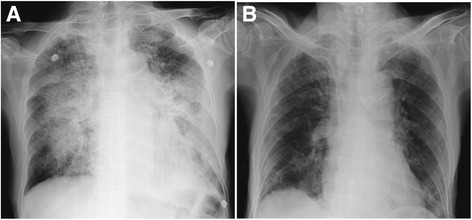
Follow-up chest X-rays. Chest X-ray at hospital day 2 showing rapid progression of ground glass opacities to consolidation (**a**). After administration of ribavirin, lesions were resolved at hospital day 27 (**b**)

**Fig. 3:**
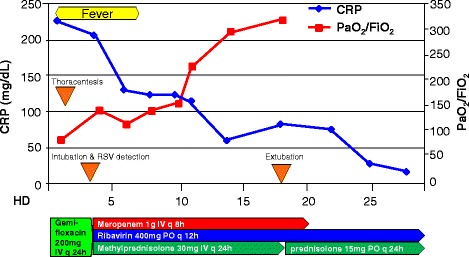
Summary of clinical course. Despite antibiotics treatment, the patient's hypoxemia deteriorated initially. After treatment with orally administered ribavirin after detection of respiratory syncytial virus, systemic inflammation and hypoxemia gradually improved. *CRP* C-reactive protein, *FiO*_*2*_ fraction of inspired oxygen, *HD* hospital day, *IV* intravenously, *PaO*_*2*_ partial pressure of oxygen in arterial blood, *PO* orally, *q* every, *RSV* respiratory syncytial virus

An 81-year-old Korean man visited our out-patient clinic complaining of cough, dyspnea, and febrile sensation. He denied any previous medical histories. He stopped smoking tobacco 30 years ago, and never drank alcohol in recent years. His vital signs were: blood pressure 140/80 mmHg, heart rate 96 beats/minute, respiratory rate 22 breaths/minute, and body temperature 38.2 °C. On physical examination, crackle was noted in both lungs. Laboratory tests revealed a white cell count of 7800/mm^3^ with slight left shift (neutrophils 88.6%), C-reactive protein (CRP) level of 223.6 mg/dL (normal < 5.0 mg/dL), total bilirubin level of 1.5 mg/dL, and alanine transaminase and aspartate transaminase levels of 59 and 61 IU/L, respectively. His sodium level was 125 mEq/mL. In arterial blood gas analysis, which was checked in ambient conditions, pH, partial pressure of carbon dioxide in arterial blood (PaCO_2_), partial pressure of oxygen in arterial blood (PaO_2_), bicarbonate, and oxygen saturation levels were 7.50, 30 mmHg, 48 mmHg, 23.4 mmol/L, and 87%, respectively. The result of a test for human immunodeficiency virus was negative. Serologic tests for *Mycoplasma* and *Chlamydia* were negative. Streptococcal and *Legionella* urinary antigens were negative. Anti-nuclear and anti-neutrophilic cytoplasmic antibodies were negative. A chest X-ray revealed diffuse haziness dominant in his right lung field (Fig. 1a). Chest computed tomography revealed ground glass opacity in both lungs with small amounts of pleural effusion dominant in the right hemithorax (Fig. 1b). With an initial assessment of community-acquired pneumonia, we administered nasal oxygen at 4L/minute and empirical antibiotics with a respiratory quinolone. At hospital day 2, thoracentesis was conducted in the right hemithorax and a turbid yellowish fluid was obtained. Pleural fluid analysis revealed lymphocyte-dominant exudate with white cell count of 560/mm^3^ and adenosine deaminase level of 4.4 IU/L. On the same day, opacities were found on chest X-ray and hypoxemia rapidly progressed to require high flow oxygen supply with fraction of inspired oxygen (FiO_2_) 0.8 at a flow rate of 40 L/minute (Fig. 2a). At hospital day 3, he had to be intubated and mechanically ventilated due to worsening hypoxemia. The initial PaO_2_/FiO_2_ after application of mechanical ventilator was 65, which was compatible with the definition of “severe” ARDS [3]. Potential cardiac dysfunction was ruled out using transthoracic echocardiography. Antibiotics were escalated to carbapenem. Multiplex real-time reverse transcriptase polymerase chain reaction (RT-PCR) was conducted using AdvanSure^TM^ respiratory virus real-time RT-PCR kit (LG Life Sciences, Seoul, Korea) to detect respiratory viruses using tracheal aspirate. Results revealed positive for human RSV type B. Under the diagnosis of RSV-induced ARDS based on the Berlin definition [4], we started antiviral therapy of orally administered ribavirin 400 mg every 12 hours with concomitant intravenously administered methylprednisolone 30 mg every 24 hours. After treatment, hypoxemia and lung lesions gradually improved. At hospital day 17, he was extubated and we tapered methylprednisolone to orally administered prednisolone 15 mg. Finally, his chest X-ray cleared and he was discharged on hospital day 27 without any complications or drug-related adverse events (Fig. 2b). Orally administered ribavirin was maintained until his discharge. We summarized the whole clinical course with the drugs administered in Fig. 3.

## Discussion

RSV pneumonia in adults occurs mostly in immunocompromised patients and is characterized by rapid clinical deterioration often leading to death. However, ARDS due to RSV in previously healthy adults is extremely rare and only two cases have been reported to date [5, 6]. Of note, patients in those cases were treated without ribavirin or with inhaled ribavirin. To the best of our knowledge, this is the first case that reports successful treatment of RSV-induced ARDS using orally administered ribavirin in an immunocompetent patient.

RSV has been considered a less significant pathogen in adults as it usually causes mild and self-limiting upper respiratory tract infection. The clinical significance of RSV infection in adults has been acknowledged in recent years. A study has estimated that RSV infects 3 to 10% of adults annually and it may be associated with 5 to 15% of community-acquired pneumonia [7]. In a retrospective cohort study, RSV infection was complicated with pneumonia in two thirds of infected patients from which one tenth required mechanical ventilation, and the mortality rate was as high as 15 to 20% comparable to that of seasonal influenza [8]. In that study, severe RSV-related lower respiratory tract infections occurred mostly in elderly patients and those with major medical comorbidities [8]. In this case, the patient denied previous medical history and had no relevant comorbidities. Therefore, the severe RSV infection may be attributable to his advanced age.

Similar to other viral diseases, RSV pneumonia is difficult to diagnose. Its respiratory symptoms are nonspecific, and laboratory and radiologic findings are usually indistinguishable from other respiratory viral infections. Definitive diagnosis of RSV can be confirmed by identification of typical plaque morphology with syncytium formation using immunofluorescent staining. However this is time consuming and costly. Nucleic acid detection using multiplex real-time RT-PCR test is used in clinical practice as it enables rapid detection with increased sensitivity [9]. In this case, atypically rapid deterioration of clinical manifestations led us to suspect infections caused by atypical pathogen including viral pneumonia. Therefore, we conducted the multiplex real-time RT-PCR test. Although diagnostic performances of different multiplex real-time RT-PCR assays for respiratory viral infections can vary depending on devices used [10, 11], the assay used for diagnosis of our case had revealed relatively high sensitivity and specificity for respiratory viral pathogens with performance that is comparable to other commercial assays despite shorter turnaround time [12]. In a previous study, Rogers *et al*. reported the clinical impact of multiplex RT-PCR test for respiratory viruses on clinical outcome using more than 1000 children patients including 344 RSV pneumonias [13]. They demonstrated that the use of a multiplex RT-PCR test was associated with decreased duration of antibiotic use, length of in-patient stay, and duration of isolation of patients admitted for acute respiratory infections. In addition, Rappo *et al*. using a retrospective cohort (*n* = 337) had compared clinical outcomes for adult patients diagnosed by multiplex RT-PCR for respiratory viruses with those diagnosed by conventional methods at a tertiary care center [14]. In that study, influenza (63% from all isolates) and RSV (15%) were the predominant viruses identified. They found a significantly lower rate of admission, length of hospital stay, duration of antimicrobial use, and number of chest radiographs, after adjusting potential confounders in the multiplex RT-PCR group [14]. Taken together, multiple RT-PCR tests are clinically useful methods for early detection of viral pathogens for respiratory tract infection and for cost effectiveness during treatment.

Our case was compatible with the Berlin definition of ARDS, defined by the timing (within 1 week of clinical insult or onset of respiratory symptoms), radiographic changes (bilateral opacities not fully explained by effusions, consolidation, or atelectasis), and origin of edema (not fully explained by cardiac failure or fluid overload). The severity of our case corresponded to “severe” [4]. There is debate about the use of corticosteroids for patients with ARDS, and current data do not support routine use of corticosteroids in those patients [3]. However, several studies suggested that low-dose corticosteroids (1 to 2 mg/kg per day of intravenously administered methylprednisolone) may be beneficial in terms of short-term mortality for patients with ARDS which is less than 14 days after onset [15, 16]. Our case was compatible with the definition of ARDS and duration of onset was less than 3 days, thus we started 30 mg of methylprednisolone intravenously. A current guideline states that a low dose of systemic steroid used in the early stage may improve hypoxemia and reduce the period of mechanical ventilation, length of intensive care unit (ICU) stay, and mortality [3]. Optimal treatment duration of corticosteroids has not been established, as corticosteroids were used for different durations at different trials. We maintained the initial dose of methylprednisolone by the time of his extubation and slowly tapered it until his discharge. The clinical benefits of corticosteroids for respiratory virus-associated ARDS as well as their optimal dose or treatment duration need to be elucidated.

Of note, pathogens other than RSV could have contributed to the development of his ARDS and a spontaneous resolution could have contributed to the outcome. However, his atypical clinical presentation and no evidence of other disease or infectious pathogen except RSV after vigorous microbiologic examination led us to suspect RSV-related ARDS. Optimal treatment for RSV pneumonia has not been established. Oral neuraminidase inhibitors have been widely used in severe influenza infection, however, they failed to show efficacy against *Paramyxoviridae* family viruses including RSV. Aerosolized ribavirin, a nucleoside analogue with broad antiviral activity, has been reported to be effective in preventing severe pneumonia in non-influenza respiratory viral infections in babies and children [17]. However, there is concern about its use due to its high cost, teratogenicity, and potentially administered risk of lung function decline. In addition, intravenously administered or aerosolized ribavirin unfortunately is not readily available in our country because we can acquire this agent only through the Korea Orphan & Essential Drug Center (KOEDC), which may cause delay in the start of treatment. Meanwhile, orally administered ribavirin is relatively safe and economic, and several reports have suggested that it is associated with favorable clinical outcomes in RSV infection [18, 19]. Thus, we selected orally administered ribavirin with systemic corticosteroids for our case. Our treatment is supported by a previous study reporting that orally administered ribavirin and corticosteroid were a well-tolerated and cost-effective regimen for lung and heart/lung transplant recipients with paramyxovirus infection [20]. This case suggests that orally administered ribavirin may be an option, although not optimal treatment, even for cases of severe RSV, especially in a situation where other forms of ribavirin are not readily available.

## Conclusions

RSV-induced ARDS is very uncommon but can be lethal in immunocompetent patients. The present case highlights the significance of early clinical suspicion and active use of multiplex real-time RT-PCR test. In addition, this case suggests that orally administered ribavirin could be a therapeutic option even for severe pneumonia or ARDS due to RSV, at least in immunocompetent hosts, especially if other antiviral agents are unavailable. Future studies are needed to determine its efficacy for selected patients.

## Abbreviations

ARDS: Acute respiratory distress syndrome
CRP: C-reactive protein
FiO_2_: Fraction of inspired oxygen
ICU: Intensive care unit
KOEDC: Korea Orphan & Essential Drug Center
PaCO_2_: Partial pressure of carbon dioxide in arterial blood
PaO_2_: Partial pressure of oxygen in arterial blood
RSV: Respiratory syncytial virus
RT-PCR: reverse transcriptase polymerase chain reaction
